# Galaxy: a comprehensive approach for supporting accessible, reproducible, and transparent computational research in the life sciences

**DOI:** 10.1186/gb-2010-11-8-r86

**Published:** 2010-08-25

**Authors:** Jeremy Goecks, Anton Nekrutenko, James Taylor

**Affiliations:** 1 Department of Biology and Department of Mathematics and Computer Science, Emory University, 1510 Clifton Road NE, Atlanta, GA 30322, USA; 2 Center for Comparative Genomics and Bioinformatics, Penn State University, 505 Wartik Lab, University Park, PA 16802, USA

## Abstract

Increased reliance on computational approaches in the life sciences has revealed grave concerns about how accessible and reproducible computation-reliant results truly are. Galaxy http://usegalaxy.org, an open web-based platform for genomic research, addresses these problems. Galaxy automatically tracks and manages data provenance and provides support for capturing the context and intent of computational methods. Galaxy Pages are interactive, web-based documents that provide users with a medium to communicate a complete computational analysis.

## Rationale

Computation has become an essential tool in life science research. This is exemplified in genomics, where first microarrays and now massively parallel DNA sequencing have enabled a variety of genome-wide functional assays, such as ChIP-seq [1] and RNA-seq [2] (and many others), that require increasingly complex analysis tools [3]. However, sudden reliance on computation has created an 'informatics crisis' for life science researchers: computational resources can be difficult to use, and ensuring that computational experiments are communicated well and hence reproducible is challenging. Galaxy helps to address this crisis by providing an open, web-based platform for performing accessible, reproducible, and transparent genomic science.

The problem of accessibility of computational tools has long been recognized. Without programming or informatics expertise, scientists needing to use computational approaches are impeded by problems ranging from tool installation; to determining which parameter values to use; to efficiently combining multiple tools together in an analysis chain. The severity of these problems is evidenced by the numerous solutions to address them. Tutorials [4,5], software libraries such as Bioconductor [6] and Bioperl [7], and web-based interfaces for tools [8,9] all improve the accessibility of computation. These approaches each have advantages, but do not offer a general solution that enables a computational tool to be easily included in an analysis chain and run by scientists without programming experience.

However, making tools accessible does not necessarily address the crucial problem of reproducibility. Reproducing experimental results is an essential facet of scientific inquiry, providing the foundation for understanding, integrating, and extending results toward new discoveries. Learning a programming language might enable a scientist to perform a given analysis, but ensuring that analysis is documented in a form another scientist can reproduce requires learning and practicing software engineering skills (Note that neither programming nor software engineering are included in a typical biomedical curriculum.) A recent investigation found that less than half of selected microarray experiments published in *Nature Genetics *could be reproduced. Issues that prevented reproduction included missing raw data, details in processing methods (especially computational ones), and software and hardware details [10]. Experiments that employ next-generation sequencing (NGS) will only exacerbate challenges in reproducibility due to a lack of standards, exceedingly large dataset sizes, and increasingly complex computational tools. In addition, integrative experiments, which use multiple data sources and multiple computational tools in their analyses, further complicate reproducibility.

To support reproducible computational research, the concept of a Reproducible Research System (RRS) has been proposed [11]. An RRS provides an environment for performing and recording computational analyses and enabling the use or inclusion of these analyses when preparing documents for publications. Multiple systems provide an environment for recording and repeating computational analyses by automatically tracking the provenance of data and tool usage and enabling users to selectively run (and rerun) particular analyses [12,13], and one such system provides a means to integrate analyses in a word-processing document [11]. While the concept of an RRS is clearly defined and well motivated, there are many open questions about what features an RRS should include and what implementation best serves the goals of reproducibility. Amongst the most important open questions are how user-generated content can be included in an RRS and how best to publish computational outputs - datasets, analyses, workflows, and tools - produced from an experiment.

Just because an analysis can be reproduced does not mean it can easily be communicated or understood. Realizing the potential of computational experiments also requires addressing the challenge of transparency: the open sharing and communication of experimental results to promote accountability and collaboration. For computational experiments, researchers have argued that computational results, such as analyses and methods, are of equal or even greater importance than text and figures as experimental outputs [14,15]. Transparency has received less attention than accessibility and reproducibility, but it may be the most difficult to address. Current RRSs enable users to share outputs in limited ways, but no RRS or other system has developed a comprehensive framework for facilitating transparency.

We have designed and implemented the Galaxy platform to explore how an open, web-based approach can address these challenges and facilitate genomics research. Galaxy is a popular, web-based genomic workbench that enables users to perform computational analyses of genomic data [16]. The public Galaxy service makes analysis tools, genomic data, tutorial demonstrations, persistent workspaces, and publication services available to any scientist that has access to the Internet [17]. Local Galaxy servers can be set up by downloading the Galaxy application and customizing it to meet particular needs. Galaxy has established a significant community of users and developers [18]. Here we describe our approach to building a collaborative environment for performing complex analyses, with automatic and unobtrusive provenance tracking, and use this as the basis for a system that allows transparent sharing of not only the precise computational details underlying an analysis, but also intent, context, and narrative. Galaxy Pages are the principal means to communicate research performed in Galaxy. Pages are interactive, web-based documents that users create to describe a complete genomics experiment. Pages allow computational experiments to be documented and published with all computational outputs directly connected, allowing readers to view the experiment at any level of detail, inspect intermediate data and analysis steps, reproduce some or all of the experiment, and extract methods to be modified and reused.

## Accessibility

**Figure 1:**
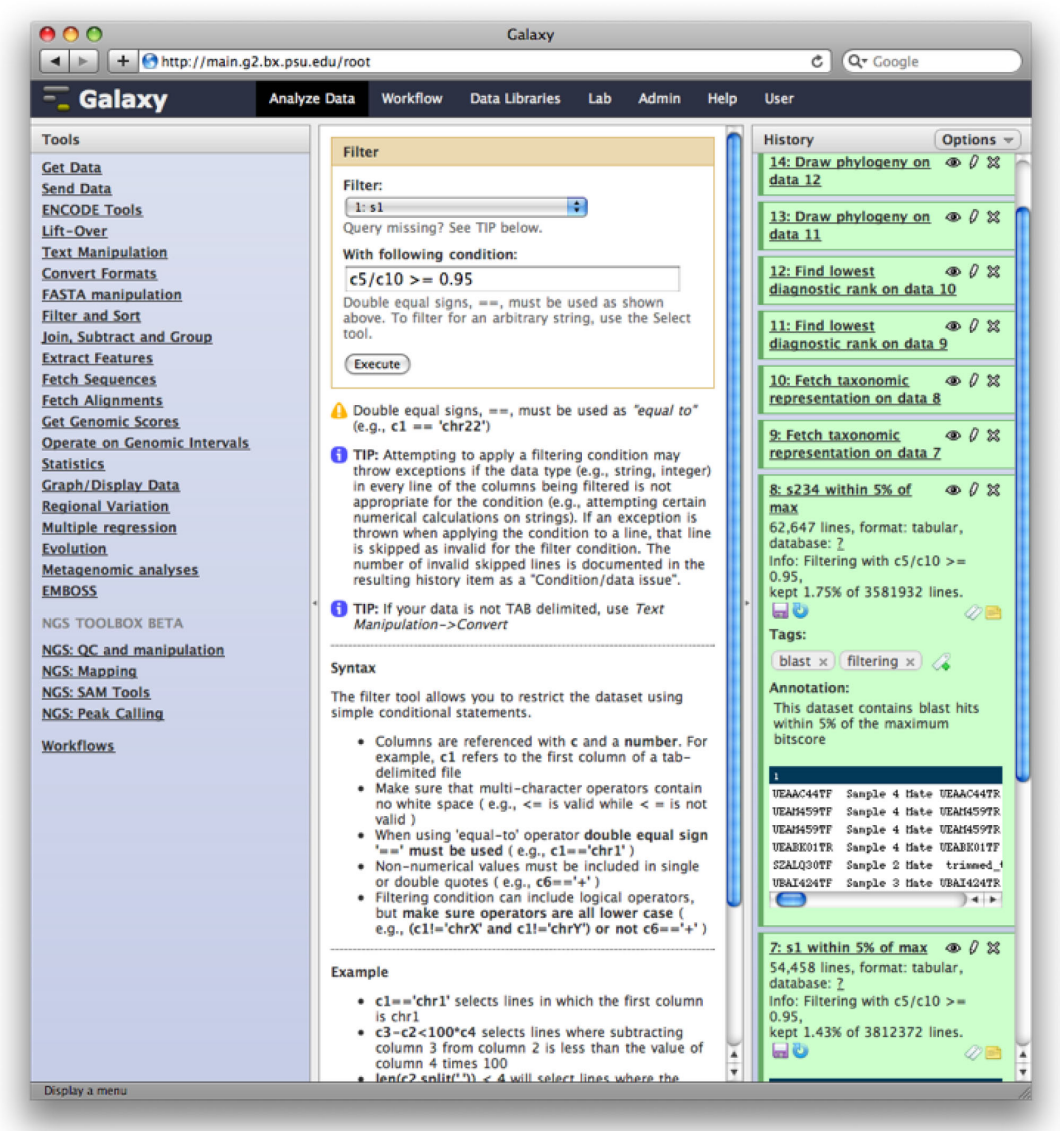
**Galaxy analysis workspace**. The Galaxy analysis workspace is where users perform genomic analyses. The workspace has four areas: the navigation bar, tool panel (left column), detail panel (middle column), and history panel (right column). The navigation bar provides links to Galaxy's major components, including the analysis workspace, workflows, data libraries, and user repositories (histories, workflows, Pages). The tool panel lists the analysis tools and data sources available to the user. The detail panel displays interfaces for tools selected by the user. The history panel shows data and the results of analyses performed by the user, as well as automatically tracked metadata and user-generated annotations. Every action by the user generates a new history item, which can then be used in subsequent analyses, downloaded, or visualized. Galaxy's history panel helps to facilitate reproducibility by showing provenance of data and by enabling users to extract a workflow from a history, rerun analysis steps, visualize output datasets, tag datasets for searching and grouping, and annotate steps with information about their purpose or importance. Here, step 12 is being rerun.

Galaxy's approach to making computation accessible has been discussed in detail in previous publications [19,20]; here we briefly review the most relevant aspects of the approach. The most important feature of Galaxy's analysis workspace is what users do not need to do or learn: Galaxy users do not need to program nor do they need to learn the implementation details of any single tool. Galaxy enables users to perform integrative genomic analyses by providing a unified, web-based interface for obtaining genomic data and applying computational tools to analyze the data (Figure 1). Users can import datasets into their workspaces from many established data warehouses or upload their own datasets. Interfaces to computational tools are automatically generated from abstract descriptions to ensure a consistent look and feel.

The Galaxy analysis environment is made possible by the model Galaxy uses for integrating tools. A tool can be any piece of software (written in any language) for which a command line invocation can be constructed. To add a new tool to Galaxy, a developer writes a configuration file that describes how to run the tool, including detailed specification of input and output parameters. This specification allows the Galaxy framework to work with the tool abstractly, for example, automatically generating web interfaces for tools as described above. Although this approach is less flexible than working in a programming language directly (for researchers that can program), it is this precise specification of tool behavior that serves as a substrate for making computation accessible and addressing transparency and reproducibility, making it ideal for command-line averse biomedical researchers.

## Reproducibility

Galaxy enables users to apply tools to datasets and hence perform computational analyses; the next step in supporting computational research is ensuring these analyses are reproducible. This requires capturing sufficient metadata - descriptive information about datasets, tools, and their invocations (that is, a number of sequences in a dataset or a version of genomic assembly are examples of metadata) - to repeat an analysis exactly. When a user performs an analysis using Galaxy, it automatically generates metadata for each analysis step. Galaxy's metadata includes every piece of information necessary to track provenance and ensure repeatability of that step: input datasets, tools used, parameter values, and output datasets. Galaxy groups a series of analysis steps into a history, and users can create, copy, and version histories. All datasets in a history - initial, intermediate, and final - are viewable, and the user can rerun any analysis step.

While Galaxy's automatically tracked metadata are sufficient to repeat an analysis, it is not sufficient to capture the intent of the analysis. User annotations - descriptions or notes about an analysis step - are a critical facet of reproducibility because they enable users to explain why a particular step is needed or important. Automatically tracked metadata record what was done, and annotations indicate why it was done. Galaxy also supports tagging (or labeling) - applying words or phrases to describe an item. Tagging has proven very useful for categorizing and searching in many web applications. Galaxy uses tags to help users find items easily via search and to show users all items that have a particular tag. Tags support reproducibility because they help users find and reuse datasets, histories, and analysis steps; reuse is an activity that is often necessary for reproducibility. Annotations and tags are forms of user metadata. Galaxy's history panel provides access to both automatically tracked metadata and user metadata (Figure 1) within the analysis workspace, and hence users can see all reproducibility metadata for a history in a single location. Users can annotate and tag both complete histories and analysis steps without leaving the analysis workspace, reducing the time and effort required for these tasks.

**Figure 2:**
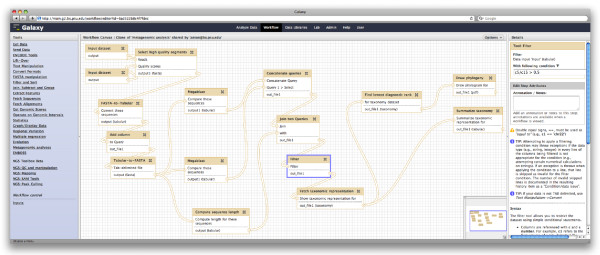
**Galaxy workflow editor**. Galaxy's workflow editor provides a graphical user interface for creating and modifying workflows. The editor has four areas: navigation bar, tool bar (left column), editor panel (middle column), and details panel. A user adds tools from the tool panel to the editor panel and configures each step in the workflow using the details panel. The details panel also enables a user to add tags to a workflow and annotate a workflow and workflow steps. Workflows are run in Galaxy's analysis workspace; like all tools executed in Galaxy, Galaxy automatically generates history items and provenance information for each tool executed via a workflow.

Recording metadata is sufficient to ensure reproducibility, but alone does not make repeating an analysis easy. The Galaxy workflow system facilitates analysis repeatability and, like Galaxy's accessibility model, in a way that is usable even to users that have little programming experience. A Galaxy workflow is a reusable template analysis that a user can run repeatedly on different data; each time a workflow is run, the same tools with the same parameters are executed. Users can also create a workflow from scratch using Galaxy's interactive, graphical workflow editor (Figure 2). Nearly any Galaxy tool can be added to a workflow. Users connect tools to form a complete analysis, and the workflow editor verifies, for each link between tools, that the tools are compatible. The workflow editor thus provides a simple and graphical interface for creating complex workflows. However, this still requires users to plan their analysis upfront. To ease workflow creation and facilitate analysis reuse, users can create a workflow by example using an existing analysis history. To develop and repeatedly run an analysis on multiple datasets requires only a few steps: 1, create and edit a history to develop a satisfactory set of analysis steps; 2, automatically generate a workflow based on the history; and 3, use the generated workflow to repeat the analysis for multiple other inputs.

A workflow is located next to all other tools in Galaxy's tool menu and behaves the same as all other tools when it is run. Workflows and all Galaxy metadata are integrated. Executing a workflow generates a group of datasets and corresponding metadata, which are placed in the current history. Users can add annotations and tags to workflows and workflow steps just as they can for histories. User annotations are especially valuable for workflows because, while workflows are abstract and can be reused in different analyses, a workflow will be reused only if it is clear what its purpose is and how it works.

## Transparency

In the course of performing analysis related to a project, Galaxy users often generate copious amounts of metadata and numerous histories and workflows. The final step for making computational experiments truly useful is facilitating transparency for the experiments: enabling users to share and communicate their experimental results and outputs in a meaningful way. Galaxy promotes transparency via three methods: a sharing model for Galaxy items - datasets, histories, and workflows - and public repositories of published items; a web-based framework for displaying shared or published Galaxy items; and Pages - custom web-based documents that enable users to communicate their experiment at every level of detail and in such a way that readers can view, reproduce, and extend their experiment without leaving Galaxy or their web browser.

**Figure 3:**
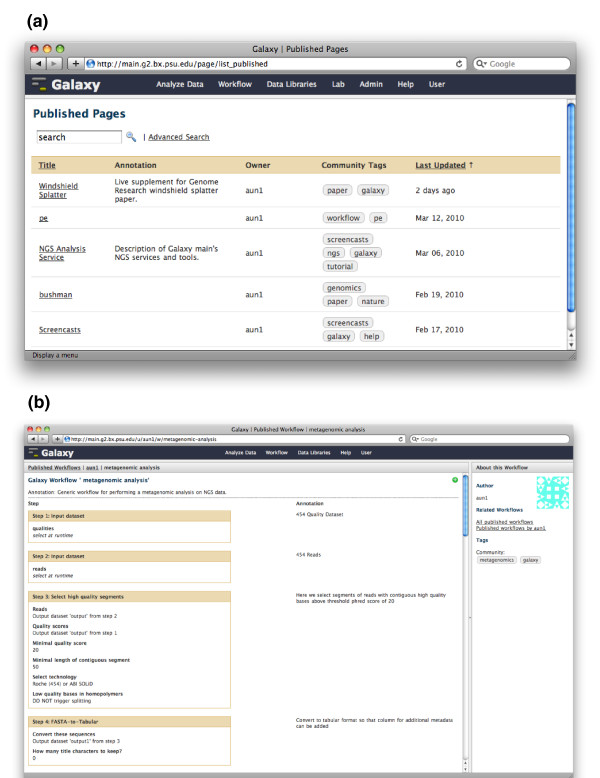
**Galaxy public repositories and published items**. **(a) **Galaxy's public repository for Pages; there are also public repositories for histories and workflows. Repositories can be searched by name, annotation, owner, and community tags. **(b) **A published Galaxy workflow. Each shared or published item is displayed in a webpage with its metadata (for example, execution details, user annotations), a link for copying the item into a user's workspace, and links for viewing related items.

Galaxy's sharing model, public repositories, and display framework provide users with means to share datasets, histories, and workflows via web links. Galaxy's sharing model provides progressive levels of sharing, including the ability to publish an item. Publishing an item generates a link to the item and lists it in Galaxy's public repository (Figure 3a). Published items have predictable, short, and clear links in order to facilitate sharing and recall; a user can edit an item's link as well. Users can search, sort, and filter the public repository by name, author, tag, and annotation to find items of interest. Galaxy displays all shared or published items as webpages with their automatic and user metadata and with additional links (Figure 3b). An item's webpage provides a link so that anyone viewing an item can import the item into his analysis workspace and start using it. The page also highlights information about the item and additional links: its author, links to related items, the item's community tags (the most popular tags that users have applied to the item), and the user's item tags. Tags link back to the public repository and show items that share the same tag.

**Figure 4:**
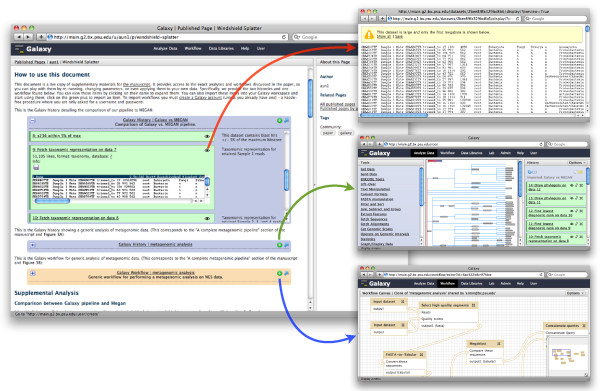
**Galaxy Pages**. Galaxy Page that is an online, interactive supplement for a metagenomic study performed in Galaxy [21]. The Page communicates all facets of the experiment via increasing levels of detail, starting with supplementary text, two embedded histories, and an embedded workflow. Readers can open the embedded items and view details for each step, including provenance information, parameter settings, and annotations. For history steps, readers can view corresponding datasets (red arrow). Readers can also copy histories (green arrow) or the workflow (blue arrow) into their analysis workspace and both reproduce and extend the experiment's analyses without leaving Galaxy or their web browser.

Galaxy Pages (Figure 4) are the principal means for communicating accessible, reproducible, and transparent computational research through Galaxy. Pages are custom web-based documents that enable users to communicate about an entire computational experiment, and Pages represent a step towards the next generation of online publication or publication supplement. A Page, like a publication or supplement, includes a mix of text and graphs describing the experiment's analyses. In addition to standard content, a Page also includes embedded Galaxy items from the experiment: datasets, histories, and workflows. These embedded items provide an added layer of interactivity, providing additional details and links to use the items as well.

Pages enable readers to understand an experiment at every level of detail. When a reader first visits a Page, he can read its text, view images, and see an overview of embedded items - an item's name, type, and annotation. Should the reader want more detail, he can expand an embedded item and view its details. For histories and workflows, expanding the item shows each step; history steps can be individually expanded as well. All metadata for both history and workflow steps are included as well. Hence, a reader can view a Page in its entirety and then expand embedded items to view every detail of every step in an experiment, from parameter settings to annotations, without leaving the Page. Currently, readers cannot discuss or comment on Pages or embedded items, though such features are planned.

Pages also enable readers to actively use and reuse embedded items. A reader can copy any embedded item into her analysis workspace and begin using that item immediately. This functionality makes reproducing an analysis simple: a reader can import a history and rerun it, or she can import a workflow and input datasets and run the workflow. Once a history or workflow is imported from a Page, a reader can also modify or extend the analysis as well or reuse a workflow in another analysis. Using Pages, readers can quickly become analysts by importing embedded items and can do so without leaving their web browser or Galaxy.

## Putting it all together: accessible, reproducible and transparent metagenomics

To demonstrate the utility of our approach, we used Pages to create an online supplement for a metagenomic study performed in Galaxy that surveyed eukaryotic diversity in organic matter collected off the windshield of a motor vehicle [21]. The choice of a metagenomic experiment for highlighting the utility of Galaxy and Pages was not accidental. Among all applications of NGS technologies, metagenomic applications are arguably one of the least reproducible. This is primarily due to the lack of an integrated solution for performing metagenomic studies, forcing researchers to use various software packages patched together with a variety of 'in-house' scripts. Because phylogenetic profiling is extremely parameter dependent - small changes in parameter settings lead to large discrepancies in phylogenetic profiles of metagenomic samples - knowing exact analysis settings are critical. With this in mind, we designed a complete metagenomic pipeline that accepts NGS reads as the input and generates phylogenetic profiles as the output.

The Galaxy Page for this study describes the analyses performed and includes the study's datasets, histories, and workflow so that the study can be rerun in its entirety [22]. To reproduce the analyses performed in the study, readers can copy the study's histories into their own workspace and rerun them. Readers can also copy the study's workflow into their workspace and apply it to other datasets without modification.

In summary, this study demonstrates how Galaxy supports the complete lifecycle of a computational biology experiment. Galaxy provides a framework for performing computational analyses, systematically repeating analyses, capturing all details of performed analyses, and annotating analyses. Using Galaxy Pages, researchers can communicate all components of an experiment - datasets, analyses, workflows, and annotations - in a web-based, interactive format. An experiment's Page enables readers to view an experiment's components at any level of detail, reproduce any analysis, and repurpose the experiment's components in their own research. All Galaxy and Page functionality is available using nothing more than a web browser.

## Galaxy usage

For the approach we have implemented in Galaxy to be successful, it must truly be usable to experimentalists with limited computational expertise. Anecdotal evidence suggests that Galaxy is usable for many biologists. Galaxy's public web server processes about 5,000 jobs per day. In addition to the public server, there are a number of high-profile Galaxy servers in use, including servers at the Cold Spring Harbor Laboratory and the United States Department of Energy Joint Genome Institute.

Individuals and groups not affiliated with the Galaxy team have used Galaxy to perform many different types of genomic research, including investigations of epigenomics [23], chromatin profiling [24], transcriptional enhancers [25], and genome-environment interactions [26]. Publication venues for these investigations include *Science*, *Nature*, and other prominent journals. Despite only recently being introduced, Galaxy's sharing features have been used to make data available from a study published in *Science *[27].

All of Galaxy's operations can be performed using nothing more than a web browser, and Galaxy's user interface follows standard web usability guidelines [28], such as consistency, visual feedback, and access to help and documentation. Hence, biologists familiar with genomic analysis tools and comfortable using a web browser should be able to learn to use Galaxy without difficulty. In the future, we plan to collect and analyze user data so that we can report quantitative measurements of how useful and usable Galaxy is for biologists and what can be done to make it better.

## Comparing Galaxy with other genomic research platforms

Accessibility, reproducibility, and transparency are useful concepts for organizing and discussing Galaxy's approach to supporting computational research. However, stepping back and considering Galaxy as a complete platform, two themes emerge for advancing computational research. One theme concerns the reuse of computational outputs, and the other theme concerns meaningful connections between analyses and sharing.

Galaxy enables reuse of datasets, tools, histories, and workflows in many ways. Automatic and user metadata make it simple for Galaxy users to find and reuse their own analysis components. Galaxy's public repository takes an initial step toward helping users publish their analysis components so that others can view and use them. Reuse is a core facet of software engineering and development, enabling large programs to be developed efficiently by leveraging past work and affording the development and sharing of best practices [29]. Enabling reuse is similarly important for life sciences computation.

Galaxy provides connections that enable users to effectively move between performing a computational experiment and publishing it. Galaxy users can annotate a history or workflow in the analysis workspace and then share an item or embed the item within a Page in just a few actions. Once shared, published or embedded, others can view the item or import it into their workspace for immediate use. Galaxy, then, makes the complete cycle of item use - from creation to annotation to publication to reuse - possible using only a web browser, making it simple for the majority of users to participate wherever in the cycle that they choose. Providing meaningful connections between analyses and publishing can encourage more publishing and a higher quality of publishing, both for Pages and for individual items. Seeing that published items are used can encourage users to publish more than they otherwise would. Well-regarded published items can serve as models for the development of other items, and hence can improve the quality of subsequently published items. Publishing, then, is closely connected with reusing analysis components.

Keeping these two themes in mind, it is useful to contrast Galaxy with other genomic workbenches to highlight Galaxy's strengths and weaknesses and suggest future directions of development for platforms supporting computational science. Currently, the most mature RRS platforms complementing Galaxy are GenePattern [12] and Mobyle [13]; both are web-based frameworks for supporting genomic research, and a primary goal of each platform is to enable reproducible research.

Table 1 summarizes Galaxy's functions and compares them with the functions of GenePattern and Mobyle. All three platforms have features that improve access to computation and facilitate reproducibility. Each platform has a unified, web-based interface for working with tools, automatically generates metadata when tools are run, and provides a framework for adding new tools to the platform. In addition, all platforms employ the concept of workflows to support repeatability. Galaxy also has features that distinguish it from both GenePattern and Mobyle. Galaxy has integrated data warehouses that enable users to employ data from these warehouses in integrative analyses. In addition, Galaxy's tags and annotations, public repository, and web-based publication framework are also unique. These features are essential for supporting both reproducibility and transparency.

**Table 1:** Comparing Galaxy to other genomic workbenches

Galaxy functionality	Description	GenePattern comparison	Mobyle comparison
**Making computation accessible**			
Unified, web-based tool interface	All tool interface share same style and use web components; tool interfaces are generated from tool configuration file	Same functions as Galaxy	Same functions as Galaxy
Simple tool integration	Tool developers can integrate tools by writing a tool configuration file and including tool file in Galaxy configuration file	Similar but not as flexible tool configuration file; easy installation of selected tools via a web-based interface	Remote services can be added using a server configuration file
Integrated datasources	Transparent access to established data warehouses	No similar functions	No similar functions
			
**Ensuring reproducibility**			
Automatic metadata	Provenance, inputs, parameters, and outputs for each tool used; analysis steps grouped into histories	Same functions as Galaxy	Same functions as Galaxy
User tags	Can apply short tags to histories, datasets, workflows, and pages; tags are searchable and facilitate reuse	No similar functions	No similar functions
User annotations	Can add descriptions or notes to histories, datasets, workflows, workflow steps, and pages to aid in understanding analyses	Cannot annotate a history but can annotate a workflow (pipeline) with an external document	No similar functions
Creating and running workflows	Can create, either by example or from scratch, a workflow that can be repeatedly used to perform a multi-step analysis	Same functions as Galaxy, although editor is form-based rather than graphical	In development
Workflow metadata	Automatic documentation is generated when a workflow is run; users can also tag and annotate workflows and workflow steps	Same functions as Galaxy for generating automatic metadata; cannot annotate workflow steps	In development
			
**Promoting transparency**			
Sharing model	Datasets, histories, workflows, and Pages can be shared at progressive levels and published to Galaxy's public repositories; datasets have more advanced sharing options, including groups	Can share analyses and workflows with individuals or groups	No similar functions
Item reuse, display framework and public repositories	Shared or published items displayed as webpages and can be imported and used immediately; public repositories can be searched; archives of analyses and workflows for sharing between servers are under development	Can create an archive of an analysis or workflow and share that with others; author information is included in archive	Can create an archive of an analysis and share that with others
Pages with embedded items	Can create custom webpages with embedded Galaxy items; each page can document a complete experiment, providing all details and supporting reuse of experiment's outputs	Microsoft Word plugin enables users to embed analyses and workflows in Word documents	No similar functions
Coupling between analysis workspace and publication workspace	Can import and immediately start using any shared, published, or embedded item without leaving web browser or Galaxy	Can run embedded analyses and save results in Microsoft Word documents	No similar functions

A summary of Galaxy's functionality and how Galaxy's functionality compares to the functionality of two other genomic workbenches, GenePattern and Mobyle. Galaxy's novel functionality includes (but is not limited to) integrated datasources, user annotations, a graphical workflow editor, Pages with embedded items, and coupling the workspaces for analysis and publication using an open, web-based model.

Perhaps the most striking difference between Galaxy and GenePattern is each platform's approach for integrating analyses and publications. Galaxy employs a web-based approach and enables users to create Pages, web-accessible documents with embedded datasets, analyses, and workflows; GenePattern provides a Microsoft Word 'plugin' that enables users to embed analyses and workflows into Microsoft Word documents.

Both approaches provide similar functions, but each platform's integration choice yields unique benefits. Galaxy's web-based approach ensures that, due to the Internet's open standards, all readers can view and interact with Galaxy Pages and embedded items. In addition, Galaxy's analysis workspace and publication workspace use the same medium, the web, and hence users can move between the two workspaces without leaving their web browser. Galaxy's publication media, webpages, matches the media used by many popular journals and hence can be used as primary or secondary documents for article submissions. The main benefit of GenePattern's Word plugin is its integration into a popular word processor that is often used for preparing articles. However, Microsoft Word documents are rarely used for archival purposes and can be difficult to view. Also, because GenePattern and Microsoft Word are two different programs, it can be difficult to move between GenePattern's analysis workspace and Word's publication workspace. These constraints limit the value of the GenePattern-Word documents.

An ideal, fully featured platform for integrating analyses and publications would likely incorporate both approaches and enable users to create both word-processing documents and webpages that share references to analyses and workflows. The ideal platform would enable users to embed objects in both a document and webpage simultaneously, synchronize a document and webpage so that changes to one are reflected in the other, and provide users with an analysis workspace accessible from either a document or a webpage. Achieving this goal will require the definition of open standards for describing and exchanging documents and analysis components between different systems, and we look forward to future developments in this direction (for example, GenomeSpace [30]).

It is also useful to compare Galaxy with other platforms that support particular aspects of genomic science and hence are complementary to Galaxy's approach. Bioconductor is an open-source software project that provides tools for analyzing and understanding genomic data [6]. Bioconductor and similar platforms, such as BioPerl [7] and Biopython [31], represent an approach to reproducibility that uses libraries and scripts built on top of a fully featured programming language. Together, Bioconductor and Sweave [32], a 'literate programming' tool for documenting Bioconductor analyses, can be used to reproduce an analysis if a researcher has the original data, the Bioconductor scripts used in the analysis, and enough programming expertise to run the scripts. Because Bioconductor is built directly on top of a fully featured programming language, it provides more flexibility and power for performing analyses as compared to Galaxy. However, Bioconductor's flexibility and power are only available to users with programming experience and hence are not accessible to many biologists. In addition, Bioconductor lacks automatic provenance tracking or a simple sharing model.

Taverna is a workflow system that supports the creation and use of workflows for analyzing genomic data [33]. Taverna users create workflows using web services and connect workflow steps using a graphical user interface much as users do when creating a Galaxy workflow. Taverna focuses exclusively on workflows; this focus makes it more difficult to communicate complete analyses in Taverna as the data must be handled outside of the system. One of Tavern's most interesting features is its use of the myExperiment platform for sharing workflows; myExperiment is a website that enables users to upload and share their workflows with others as well as download and use others' workflows [34].

Both Bioconductor and Taverna offer features that complement Galaxy's functionality. Galaxy's framework can accommodate Bioconductor's tools and scripts without modification; to integrate a Bioconductor tool or script, all a developer needs to do is write a tool definition file for it. We are actively working to integrate Galaxy's workflow sharing functionality with myExperiment so that Galaxy workflows can be shared via myExperiment.

## Future directions and challenges

Galaxy's future directions arise from efforts to balance support for cutting-edge genomic science with support for accessible, reproducible, and transparent science. The increasingly large size of many datasets is one particularly challenging aspect of current and future genomic science; it is often prohibitive to move large datasets due to constraints in time and money. Hence, local Galaxy installations near the data are likely to become more prevalent because it makes more sense to run Galaxy locally as compared to moving the data to a remote Galaxy server.

Ensuring that Galaxy's analyses are accessible, reproducible, and transparent as the number of Galaxy servers grows is a significant challenge. It is often difficult to provide easy and persistent access to Galaxy analyses on a local server; easy access is necessary for collaborative work, and persistent access is needed for published analyses. Local servers are often difficult to access (for example, if it is behind a firewall), and additional work is often needed to ensure that a local server is functioning well.

We are pursuing three strategies to ensure that any Galaxy analysis and associated objects can be made easily and persistently accessible. First, we are developing export and import support so that Galaxy analyses can be stored as files and transferred among different Galaxy servers. Second, we are building a community space where users can upload and share Galaxy objects. Third, we plan to enable direct export of Galaxy Pages and analyses associated with publications to a long-term, searchable data archive such as Dryad [35].

Local installations also pose challenges to Galaxy's accessibility because it can be difficult to install tools that Galaxy runs. Using web services in Galaxy would reduce the need to install tools locally; many large life sciences databases, such as BLAST [9] and InterProScan [36], provide access via a programmatic web interface. However, web services can compromise the reproducibility of an analysis because a researcher cannot determine or verify details of the program that is providing a web service. Also, a researcher cannot be assured that a needed web service will be available when trying to reproduce an analysis. Because web services can significantly compromise reproducibility, they are not a viable approach for use in Galaxy.

A related problem is how best to enable researchers to install and choose which version of a tool to run. Galaxy's metadata include the version of each tool run, but this information is not yet exposed to users. We are extending the Galaxy framework to support simultaneously integrating tools that require different versions of an underlying program or library. To ease the burden of installing and administering tool dependencies, we are pursuing the approach of building virtual machine images that can be used to deploy a personal Galaxy server locally or on a 'cloud' computing resource with particular tool suites (and tool versions) included.

Finally, increasing the choices that researchers have when installing and using Galaxy leads to a new challenge. Requiring a user to select tool suites during installation and tool versions and parameters during analysis can be problematic; presenting users with so many choices can lead to confusion or require users to make choices that they are unsure of. Workflows provide one solution to this problem, by predefining parameters and ways of composing tools for specific types of analysis. To help users make better and faster choices within Galaxy, we are extending Galaxy's sharing model to help the Galaxy user community find and highlight useful items. Ideally, the community will identify histories, workflows, and other items that represent best practices; best practice items can be used to help guide users in their own analyses.

We have proposed a model for a reproducible research system based on three qualities: accessibility, reproducibility, and transparency. Galaxy implements this model using a web-based, open framework, and users can access all of Galaxy's features using only a standard web browser. Galaxy Pages draw together much of Galaxy's functionality to provide a new publishing method. Galaxy Pages enable biologists to describe their experiments using web-based documents that include embedded Galaxy objects. An experiment's Page communicates all facets of the experiment via increasing levels of detail and enables readers to reproduce the experiment or reuse the experiment's methods without leaving Galaxy. The life sciences community has used Galaxy to perform analyses that contributed to numerous publications, and we have used Galaxy Pages to provide supplementary material for a published metagenomics experiment. In the future, large datasets and increasing access to computation likely means that more biologists will have access to a personal Galaxy server. A main challenge for Galaxy is continuing to enable accessible, reproducible, and transparent genomic science while also facilitating more personal and distributed access to Galaxy's functionality.

## Details of Galaxy Framework and selected features

The Galaxy Framework is a set of reusable software components that can be integrated into applications, encapsulating functionality for describing generic interfaces to computational tools, building concrete interfaces for users to interact with tools, invoking those tools in various execution environments, dealing with general and tool-specific dataset formats and conversions, and working with 'metadata' describing datasets, tools, and their relationships. The Galaxy Application is an application built using this framework that provides access to tools through an interface (for example, a web-based interface) and provides features for performing reproducible computational research as described in this paper. A Galaxy server, or Instance, is a deployment of this application with a specific set of tools.

Galaxy is implemented primarily in the Python programming language (tested on versions 2.4 through 2.6). It is distributed as a standalone package that includes an embedded web server and SQL (structured query language) database, but can be configured to use an external web server or database. Regular updates are distributed through a version control system, and Galaxy automatically manages database and dependency updates. A Galaxy instance can utilize compute clusters for running jobs, and can be easily interfaced with portable batch system (PBS) or Sun Grid Engine (SGE) clusters.

The editors for tagging and annotations are integrated into Galaxy's analysis workspace and are designed to support web-based genomic research. Galaxy tags are hierarchical and can have values, and these features make tags amenable to many different metadata vocabularies and navigational techniques. For instance, the tag encode.cell_line = K562 indicates that the item uses Encode K562 cell line; the tag is 'encode.cell_line,' and its value is 'K562.' Using this tag, Galaxy can find all items that have this tag and value (encode.cell_line = K562), all items that have this tag, regardless of value (encode.cell_line), or all items that share a parent tag (encode or encode. < anything >). We are currently developing an interface for browsing tagged items. We are also implementing item tags for datasets stored in Galaxy libraries; this is especially useful because Galaxy libraries are repositories for shared datasets, and helping researchers find relevant libraries and library datasets is often difficult. Users can style their annotations (for example, use bold and italics) and add web links to them. Because annotations are displayed on webpages via Galaxy's publication framework, it makes sense that users are able to take advantage of the fact that annotations are displayed on webpages.

Galaxy's workflow editor provides an interactive graphical interface that enables users to visually build and connect tools to create workflow. A user can add a box to represent any of the tools in Galaxy's tool panel (with the exception of several datasources access tools at the time of writing) to the workflow editor canvas. The user then connects tools to create a flow of data from one tool to the next and ultimately an analysis chain; connecting tools is done by dragging links from one tool to another. The workflow editor can determine which tools can be chained together: if the output of tool A is compatible with the input of tool B, these two can be chained together. Valid links between tools are green, and invalid links are red.

Galaxy's sharing model provides three progressive levels of sharing. First, a user can share an item with other users. Second, a user can make an item accessible; making an item accessible generates a web link for the item that a user can share with others. Unlike when an item is shared with other users, an accessible item can be viewed by anyone that knows the item's link, including non-Galaxy users. Third, a user can publish an item; publishing an item makes the item accessible and lists the item in Galaxy's public repository. Accessible or published items have consistent, clear links that employ the item owner's public username, the item type, and the item identifier. For instance, an accessible history owned by a user with the username 'jgoecks' and using the identifier 'taf1-microarray-analysis' would have the relative URL /jgoecks/h/taf1-microarray-analysis Galaxy item links are simple in order to facilitate sharing and recall; a user can edit an item's identifier as well and hence change its URL. Sharing an item and editing its identifier are done through a simple web-based interface.

Galaxy's Page editor looks and feels like a word processing program. The editor enables a Galaxy user to create a free-form web document using text, standard web components (for example, images, links, tables), web styles (for example, paragraphs, headings) and embedded Galaxy items. Embedding Galaxy items is done via standard lists and buttons, and embedded Galaxy items look like colored blocks in the text when a user is editing a Page. The embedding framework is sufficiently general to allow other types of items, such as visualizations and data libraries, to be embedded in Pages in the future.

## Abbreviations

NGS: next-generation sequencing; RRS: reproducible research system.

## Authors' contributions

JG, AN, and JT designed the approach, collected results, and wrote the manuscript. JG, AN, JT, and the Galaxy team implemented the Galaxy framework and maintain its public instance.
